# Differential Impact of miR-21 on Pain and Associated Affective and Cognitive Behavior after Spared Nerve Injury in B7-H1 ko Mouse

**DOI:** 10.3389/fnmol.2017.00219

**Published:** 2017-07-11

**Authors:** Franziska Karl, Anne Grießhammer, Nurcan Üçeyler, Claudia Sommer

**Affiliations:** Department of Neurology, University of Würzburg Würzburg, Germany

**Keywords:** B7-H1, PD-L1, immune system, neuropathic pain, SNI, miRNA, miR-21

## Abstract

MicroRNAs (miRNAs) are increasingly recognized as regulators of immune and neuronal gene expression and are potential master switches in neuropathic pain pathophysiology. miR-21 is a promising candidate that may link the immune and the pain system. To investigate the pathophysiological role of miR-21 in neuropathic pain, we assessed mice deficient of B7 homolog 1 (B7-H1), a major inhibitor of inflammatory responses. In previous studies, an upregulation of miR-21 had been shown in mouse lymphocytes. Young (8 weeks), middle-aged (6 months), and old (12 months) B7-H1 ko mice and wildtype littermates (WT) received a spared nerve injury (SNI). We assessed thermal withdrawal latencies and mechanical withdrawal thresholds. Further, we performed tests for anxiety-like and cognitive behavior. Quantitative real time PCR was used to determine miR-21 relative expression in peripheral nerves, and dorsal root ganglia (DRG) at distinct time points after SNI. We found mechanical hyposensitivity with increasing age of naïve B7-H1 ko mice. Young and middle-aged B7-H1 ko mice were more sensitive to mechanical stimuli compared to WT mice (young: *p* < 0.01, middle-aged: *p* < 0.05). Both genotypes developed mechanical and heat hypersensitivity (*p* < 0.05) after SNI, without intergroup differences. No relevant differences were found after SNI in three tests for anxiety like behavior in B7-H1 ko and WT mice. Also, SNI had no effect on cognition. B7-H1 ko and WT mice showed a higher miR-21 expression (*p* < 0.05) and invasion of macrophages and T cells in the injured nerve 7 days after SNI without intergroup differences. Our study reveals that increased miR-21 expression in peripheral nerves after SNI is associated with reduced mechanical and heat withdrawal thresholds. These results point to a role of miR-21 in the pathophysiology of neuropathic pain, while affective behavior and cognition seem to be spared. Contrary to expectations, B7-H1 ko mice did not show higher miR-21 expression than WT mice, thus, a B7-H1 knockout may be of limited relevance for the study of miR-21 related pain.

## Introduction

Lesions or diseases of the somatosensory nervous system may lead to neuropathic pain (Treede et al., 2008), which is a major burden to the afflicted patients, and may entail depression and anxiety (Jain et al., 2011). Neuropathic pain has a strong negative impact on everyday life activities and working performance and immensely reduces patients’ health related quality of life (Blyth et al., 2003). Neuro-immune interactions may be crucial for the development of neuropathic pain (Ellis and Bennett, 2013), and similar pathomechanisms have been implied for depression (Furtado and Katzman, 2015). Interactions between pro- and anti-inflammatory systems seem to play a major role (Üçeyler et al., 2009), which is underlined by data on disturbed pain behavior in animal models of cytokine or immune component deficiency (Cunha et al., 1999; Vale et al., 2003; Schoeniger-Skinner et al., 2007; Karam et al., 2011; Üçeyler et al., 2011; Sun et al., 2016).

B7 homolog 1 (B7-H1; synonyms: PD-L1, CD274) is a type 1 transmembrane protein and a member of the B7/CD28 family (Dong et al., 1999; Ostrand-Rosenberg et al., 2014). B7-H1 was identified as a ligand for the programmed-death receptor-1 (PD-1) and its interaction with the B7-H1 receptor dampens T cell proliferation and cytokine production. Using the model of chronic constrictive nerve injury (CCI), we have previously shown that B7-H1 deficiency leads to an excessive pro-inflammatory response and prolonged and enhanced pain behavior after peripheral nerve lesion (Üçeyler et al., 2010). Others reported that intraplantar injection of B7-H1 causes analgesia in an inflammatory mouse model (Chen et al., 2017). These results propose B7-H1 as an interesting candidate to study neuropathic pain pathomechanisms and potentially associated symptoms of depression and anxiety.

MicroRNAs (miRNAs; 19–25 nucleotides) negatively regulate gene expression by translational inhibition or degradation of the targeted mRNA (Ha and Kim, 2014). Since the miRNA-mRNA functional pairing does not need a fully complementary sequence, miRNAs are able to bind to several mRNAs and thus can simultaneously influence multiple mediators in different pathways (Lewis et al., 2005; Bali and Kuner, 2014). Their potential to regulate neuronal and immune gene expression simultaneously makes miRNAs promising candidates for master switches that might be of particular importance in neuropathic pain pathophysiology (Soreq and Wolf, 2011; Wanet et al., 2012). Indeed, recent research has identified miRNAs that are crucially involved in pain pathways (Kusuda et al., 2011; Yu et al., 2011; Lin et al., 2014), including miRNA-21 (miR-21). miR-21 controls pro- and anti-inflammatory responses in leukocytes and non-hematopoietic cells (Sheedy, 2015), it was upregulated in the dorsal root ganglia (DRG) of neuropathic rats after spinal nerve ligation and neuropathic pain behavior was relieved by intrathecal administration of a miR-21 inhibitor (Sakai and Suzuki, 2013). An upregulation of miR-21 has been shown in T cells of PD-1 ko mice (Iliopoulos et al., 2011). We thus hypothesized that the lack of B7-H1 together with miR-21 upregulation might determine the pain phenotype in B7-H1 knock-out (ko) mice. We used the spared nerve injury (SNI) model to analyze a lesioned and an unlesioned nerve branch separately for potential changes in miR-21 expression.

## Materials and Methods

### Ethics Statement

All experiments were approved by the Bavarian State authorities (Regierung von Unterfranken, #3/12). Mice were housed in the animal facilities of the University of Würzburg (Department of Neurology, Center for Experimental Molecular Medicine) with food and water access *ad libitum*. Animal use and care were in accordance with the institutional guidelines.

### Animals

A total of 290 male and female mice (188:102) were investigated. B7-H1 ko mice were generated by L. Chen, Baltimore, MD, USA (Dong et al., 2004), breeding pairs were supplied to us, and inbred wild-type littermates (WT) of C57Bl/6J background served as controls.

### Behavioral Testing

All behavioral tests were performed by an experienced investigator (FK) blinded to the genotype. We examined several age groups due to known age-dependent alterations in pain, affective and cognitive behavior (Crisp et al., 2003; Lemmer et al., 2015; Shoji et al., 2016; Üçeyler et al., 2016). For mechanical and thermal sensitivity mice of three age-groups were investigated: young (8 weeks), middle-aged (6 months), and old (12 months) mice. For affective and cognitive behavior we concentrated on young and old mice.

#### Mechanical and Thermal Sensitivity

To obtain baseline values and allow the animals to adapt to the testing apparatus, all animals were tested three times before surgery. After SNI (see below), behavioral tests were performed at designated time points (3, 7 and 14 days after surgery, *n* = 6 mice/genotype and age-group). The von-Frey test based on the up-and-down-method was used to investigate the paw withdrawal thresholds upon mechanical stimulation (Chaplan et al., 1994). Animals were placed in plexiglass cages on a wire mesh. After 45 min of adaptation, the lateral plantar surface of the hind paws (i.e., sural nerve innervation territory) was touched with a von-Frey filament starting at 0.69 g. If the mouse withdrew its hind paw, the next finer von-Frey filament was used. If the mouse did not show any reaction, the next thicker von-Frey filament was applied. Each hind paw was tested six times. The 50% withdrawal threshold (i.e., force of the von-Frey hair to which an animal reacts in 50% of the administrations) was calculated.

To determine the sensitivity to thermal heat stimuli we used the Hargreaves method applying a standard Ugo Basile Algesiometer (Comerio, Italy; Hargreaves et al., 1988). Mice were placed on a glass surface. After a 45 min adaption period, a radiant heat stimulus (25 IR) was applied to the lateral plantar surface of the hind paw and the withdrawal latency was automatically recorded. To prevent tissue damage by heat, we used a stimulus cutoff time of 16 s. Each hind paw was consecutively tested three times.

Paw withdrawal latencies to cold stimuli were determined using the cold plantar test (Brenner et al., 2012). Mice were placed in plexiglass cages on a glass surface (1/4″) and a dry ice stick was applied against the glass at the lateral plantar side of the hind paw (i.e., sural nerve innervation territory). Time until paw withdrawal was recorded with a maximum time limit for stimulus application of 20 s to avoid tissue damage.

#### Tests for Affective and Cognitive Behavior

Mice were housed in a reversed light-dark cycle (light cycle: 7 p.m.–7 a.m.; dark cycle: 7 a.m.–7 p.m.) and were tested during their active phase under infra-red light. Behavioral tests were performed in a black box, to avoid interference with other mice and the investigator. All tests were video recorded for further analysis (see below).

##### Anxiety- and depression-like behavior

We performed three different tests for anxiety-like behavior: light-dark box (LDB; Crawley and Goodwin, 1980), elevated plus maze (EPM; Pellow et al., 1985), and open field (OF; Prut and Belzung, 2003) to assess the intra-individual variation in affective behavior. EPM and OF were also used to investigate exploratory behavior of the mice. Each mouse was tested once for 5 min in each apparatus.

The LDB consisted of an illuminated (40 cm × 20.5 cm) and a dark compartment (40 cm × 19.5 cm). Each mouse was first placed into the lit box. Mice could freely explore the apparatus and choose between the two inter-connected compartments. The percentage of time spent in the dark box was recorded.

The EPM apparatus consisted of two opposite open arms (66.5 cm) and two closed arms (65.5 cm), separated by a junction area. Mice were placed individually in the middle of the apparatus, facing an open arm. The total time spent in closed arms, the entries into open arms, and the total distance traveled were determined.

The OF (40 cm × 40 cm) consisted of two areas: the center zone (20 cm × 20 cm) and the surrounding area. Mice were individually placed in the middle of the center zone; time spent in the center zone, the total distance traveled and the average speed were recorded.

To test for depression-like behavior we performed the forced-swim test (FST; Porsolt et al., 1977). Mice were placed in a glass cylinder, filled with water (diameter of cylinder: 11.5 cm; water height: 12.5 cm; water temperature: 20°C ± 2°C). Time spent immobile during 5 min of observation was recorded within a 6 min testing phase.

##### Cognitive behavior

The Morris water maze (MWM) test (Morris, 1984) was used to investigate learning behavior and memory. Tests were performed in a cylindrical plastic pool (diameter: 118.5 cm), filled with opaque water (temperature: 20°C ± 2°C) just covering the platform (diameter: 8 cm). The pool was divided into four quadrants and the platform was placed in the south-east quadrant (= target quadrant). Mice had four daily trails with different starting points (located in the middle of each quadrant) on four consecutive training days. We measured the time mice needed to reach the platform and calculated the daily average time for every group. Mice that did not find the platform within 60 s were placed on the platform for 15 s for orientation in the pool. On the 5th day, we performed the probe trial (PT) for memory performance, during which the platform was removed. For the PT a new starting point on the opposite side of the target quadrant was chosen. Time mice spent in the target quadrant, the total distance they traveled and the average speed was measured during a 30 s observation period.

### Spared Nerve Injury (SNI)

Naïve mice were anesthetized with isoflurane (2% induction, 1.5% maintenance) in a 50% O_2_/room air mixture. SNI or a sham surgery was performed as described (Decosterd and Woolf, 2000). Skin on the lateral surface of the right thigh was incised and the sciatic nerve and its three branches were exposed by a blunt dissection through the biceps femoris muscle. Distal to the trifurcation of its branches, the common peroneal and the tibial nerves were ligated (using 7.0 silk) and axotomized, removing a 2–4 mm piece of each distal nerve stump. Care was taken to keep the sural nerve untouched. Incisions were closed with muscle and skin sutures. In sham surgery, the sciatic nerve branches were exposed, but not injured. Behavioral experiments were conducted 3–21 days after surgery.

### Tissue Collection

At the end of the experiments, mice were sacrificed in deep isoflurane anesthesia and the ipsilateral nerve stump (common peroneal and tibial nerve) of the sciatic nerve (proximal to the nerve injury), the sural nerve, and the L4 and L5 DRG were dissected for quantitative real-time-PCR (qRT-PCR) using a dissection microscope (Zeiss, Oberkochen, Germany) at the following time points: baseline, 5, 7, 15 days after SNI (*n* = 6 mice/age-group). Tissue from naïve mice served as controls. Tissue was shock frozen in liquid nitrogen and was stored at −80°C before further processing. For immunohistochemistry the ipsilateral nerve stump (common peroneal and tibial nerve) of the sciatic nerve and the sural nerve (*n* = 5 per group) were dissected 7 days after SNI. Tissue was embedded in Tissue Tek, optimal cutting temperature (OCT) medium (Sakura, Staufen, Germany), frozen in 2-methylbutane cooled in liquid nitrogen and stored at −80°C until further processing.

### qRT-PCR Studies

Total RNA isolation from dissected tissue was performed using the miRNeasy Micro Kit (Qiagen, Hilden, Germany). RNA concentration was quantified spectrophotometrically with the NanoPhotometer Pearl^®^ (Implen, Munich, Germany).

TaqMan qRT-PCR (Applied Biosystems, Darmstadt, Germany) was used for gene expression analysis of B7-H1 levels (Cd274, Assay ID: Mm03048248_m1). PCR reagents were used from Life Technologies (Carlsbad, CA, USA). RNA (150 ng) was reversed transcribed using TaqMan Reverse Transcription Reagents, following manufacturer’s protocol. For each sample 5 μl cDNA was applied. 18sRNA (Assay ID: Hs99999901_s1) was used as an endogenous control and data were assessed using the ΔΔCt method.

For miRNA-specific synthesis of first strand cDNA, 5 ng of total RNA was transcribed applying the Universal cDNA Synthesis kit II (Exiqon, Vedbaek, Denmark) following the manufacturer’s recommendations. For each reaction 4 μl of diluted (1:80) cDNA was PCR amplified applying the corresponding miRNA and reference primer sets, using the miCURYLNA^TM^ Universal microRNA PCR (Exiqon, Vedbaek, Denmark) and following the manufacturer’s protocol. The expression levels of miR-21–5p (5′-3′ TAGCTTATCAGACTGATGTTGA) were normalized to the expression of the endogenous control Sno202 (5′-3′ GCTGTACTGACTTGATGAAAGTACTTTTGAACCCTTTTCCATCTGATG). Sno202 was chosen as an endogenous control for individual target normalization since it showed the most stable qRT-PCR results among several different candidates tested (U5, Snord65, U1A1, 5S, Snord110, RNU5G, Sno234, Sno202). miRNA-21 was amplified in triplicate and threshold cycle (Ct) values were obtained. Fold changes in miRNA expression among groups were calculated using the ΔΔCt method.

### Immunohistochemistry

Ten-micrometer cryosections of the common peroneal/tibial nerve and the sural nerve were prepared using a cryostat (Leica, Blenheim, Germany). Immunohistochemical staining with antibodies against CD11b (rat, 1:250, Serotec, MCA711, Puchheim, Germany) for the detection of monocytes/macrophages (further referred to in the text as “macrophages”) and CD 3 (rat, 1:100, Serotec, MCA1477, Puchheim, Germany) for the detection of T cells were performed following standard methods using 0.02% diaminobenzidine (DAB) as chromogen and hemalaun as a counter staining. Anti-rat IgG were used as secondary antibodies. On negative control sections the primary antibody was omitted. Images were acquired using an Axiophot 2 microscope (Zeiss, Oberkochen, Germany) equipped with a CCD camera (Visitron Systems, Tuchheim, Germany). Immunopositive profiles were quantified manually in at least three sections for each mouse and related to the area of the sections. Data were analyzed using SPOT software (software version 5.2, Spot Software BV, Amsterdam, Netherlands) and ImageJ free software version 1.51f (National Institute of Health, Staten Island, NY, USA).

### Video Processing and Statistical Analysis

Recorded videos were analyzed using the ANY-maze video tracking software (system version: 4.99 m, Stoelting, Wood Dale, IL, USA). For statistical analysis and graph design SPSS IBM software Version 23 was employed (Ehningen, Germany). The non-parametric Mann-Whitney U test was applied, since data were not normally distributed in the Kolmogorov-Smirnov test. The Bonferroni-Holm procedure was applied to correct for multiple comparisons as appropriate. Data are illustrated as box plots, bar graphs or line charts as appropriate (SPSS IBM software version 23 and GraphPad Prism, software version 5.03, San Diego, CA, USA). Data were stratified for age (young: 8 weeks, middle-aged: 6 months, old: 12 months) and treatment groups (naïve, sham, SNI). *P* values < 0.05 were considered statistically significant. Data of the qRT-PCR are illustrated as box plots, representing the median value and the upper and lower 25% and 75% quartile. All other data were expressed in bar graphs or line charts as mean with standard error of the mean (SEM).

## Results

### Mechanical and Heat Hypersensitivity in B7-H1 ko and WT Mice after SNI

**Figure 1:**
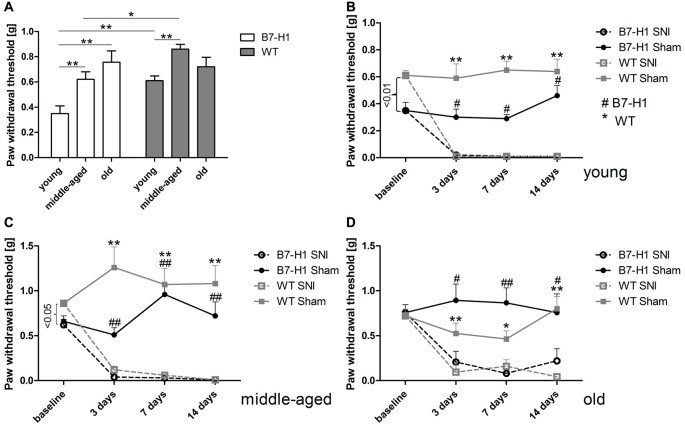
Paw withdrawal thresholds to mechanical stimulation. Bar graph and line charts show the results of the von-Frey test in young (8 weeks), middle-aged (6 months), and old (12 months) B7-H1 knock-out (ko) and wild-type littermate (WT) mice at baseline and 3, 7 and 14 days after spared nerve injury (SNI) or sham surgery. **(A)** Naïve young (*p* < 0.01) and middle-aged (*p* < 0.05) B7-H1 ko mice showed mechanical hypersensitivity compared to WT mice. At baseline young **(B)** and middle-aged **(C)** B7-H1 ko mice displayed lower mechanical thresholds compared to WT mice (*p* < 0.01, *p* < 0.05). Old **(D)** B7-H1 ko and WT mice did not differ in mechanical perception at baseline. Young, middle-aged and old B7-H1 ko and WT mice developed mechanical hypersensitivity 3 days (young: B7-H1 ko *p* < 0.05, WT *p* < 0.01; middle-aged: B7-H1 ko and WT *p* < 0.01 each; old: B7-H1 ko *p* < 0.05, WT *p* < 0.01), 7 days (young: B7-H1 ko *p* < 0.05, WT *p* < 0.01; middle-aged: B7-H1 ko and WT *p* < 0.01 each; old: B7-H1 ko *p* < 0.01, WT *p* < 0.05), and 14 days (young: B7-H1 ko *p* < 0.05, WT *p* < 0.01; middle-aged: B7-H1 ko and WT *p* < 0.01 each; old: B7-H1 ko *p* < 0.05, WT *p* < 0.001) after SNI without intergroup differences. Mechanical withdrawal thresholds of sham operated B7-H1 ko and WT mice did not differ from baseline values 3, 7 and 14 days after surgery. B7-H1 ko: young (8 weeks; SNI: 4 males, 2 females; sham: 2 males, 2 females), middle-aged (6 months; SNI: 3 males, 3 females; sham: 3 males, 3 females), old (12 months, SNI: 3 males, 5 females; sham: 3 males, 3 females). WT: young (8 weeks; SNI: 3 males, 3 females; sham: 3 males, 3 females), middle-aged (6 months; SNI: 3 males, 3 females; sham: 3 males, 3 females), old (12 months, SNI: 3 males, 5 females; sham: 3 males, 4 females). *^,^^#^*p* < 0.05; **^,^^##^*p* < 0.01.

**Figure 2:**
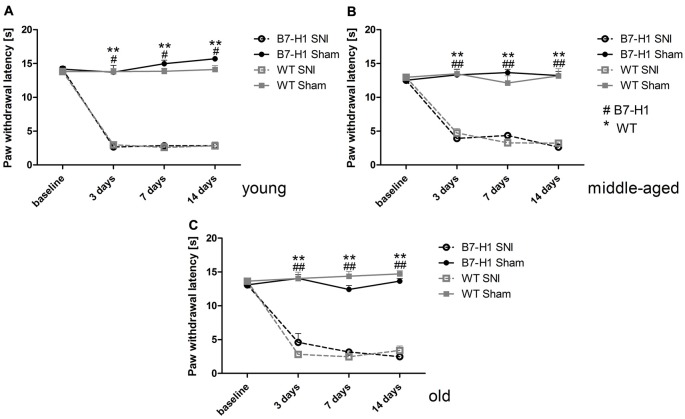
Paw withdrawal latencies to heat stimulation. Line charts show the results of the Hargreaves test in young (8 weeks), middle-aged (6 months), and old (12 months) B7-H1 ko and wild-type littermate (WT) mice at baseline and 3, 7 and 14 days after SNI or sham surgery. At baseline young **(A)** middle-aged **(B)** and old **(C)** B7-H1 ko and WT mice did not differ for paw withdrawal latencies to heat stimulation. Young, middle-aged and old B7-H1 ko and WT mice developed hypersensitivity to heat stimuli 3, 7 and 14 days after SNI without intergroup differences (young B7-H1 ko: *p* < 0.05, young WT, middle-aged B7H1 ko and WT, old B7-H1 ko and WT: *p* < 0.01). Sham operated B7-H1 ko and WT mice did not show differences 3, 7 and 14 days after surgery. B7-H1 ko: young (8 weeks; SNI: 4 males, 2 females; sham: 2 males, 2 females), middle-aged (6 months; SNI: 3 males, 3 females; sham: 3 males, 3 females), old (12 months, SNI: 3 males, 5 females; sham: 3 males, 3 females). WT: young (8 weeks; SNI: 3 males, 3 females; sham: 3 males, 3 females), middle-aged (6 months; SNI: 3 males, 3 females; sham: 3 males, 3 females), old (12 months, SNI: 3 males, 5 females; sham: 3 males, 4 females). ^#^*p* < 0.05; **^,^^##^*p* < 0.01.

Naïve young (*p* < 0.01) and middle-aged (*p* < 0.05) B7-H1 ko mice showed mechanical hypersensitivity compared to WT mice, while mechanical thresholds were normal in old B7-H1 ko mice (Figure 1A). After SNI, young, middle-aged and old B7-H1 ko and WT mice developed mechanical hypersensitivity that was already detectable on day 3 after surgery (young B7-H1 ko: *p* < 0.05, young WT: *p* < 0.01, middle-aged B7-H1 ko and WT: *p* < 0.01 each, old B7-H1 ko: *p* < 0.05, old WT: *p* < 0.01, Figures 1B–D) without differences between genotypes. Heat withdrawal latencies did not differ between genotypes at baseline in any age-group, and both genotypes developed heat hypersensitivity after SNI without intergroup differences (young B7-H1 ko: *p* < 0.05, young WT, middle-aged and old B7-H1 ko and WT *p* < 0.01 each, Figure 2). Cold withdrawal latencies were not different at baseline and after SNI between genotypes and age-groups (data not shown).

### No Influence of SNI on Anxiety-Like Behavior

**Figure 3:**
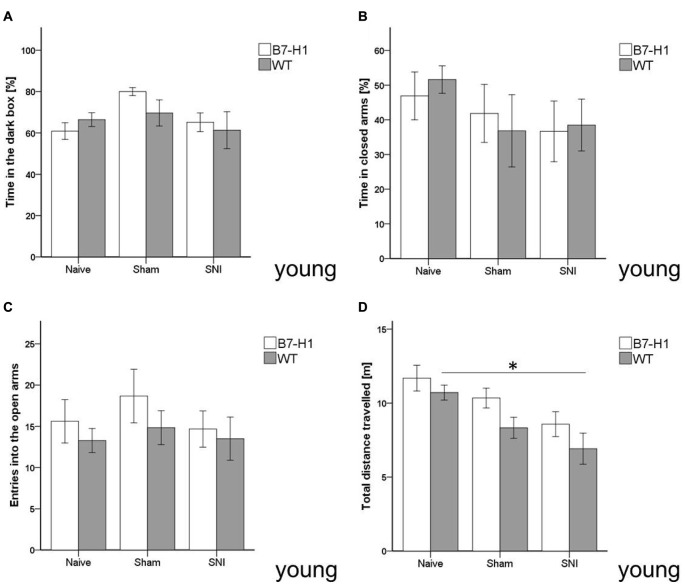
Anxiety-like behavior and locomotor activity in the elevated plus maze (EPM) and light-dark box (LDB). Bar graphs show the results of the EPM and LDB in 8 weeks old, male B7-H1 ko and WT littermates. Mice were investigated in the naïve state, after sham surgery and after SNI. No differences were detected in the time naïve B7-H1 ko mice spent in the dark box of the LDB **(A)** and in the closed arms of the EPM **(B)** compared to WT mice. SNI did not lead to differences in time spent in the dark box and closed arms compared to naïve mice. There was also no intergroup difference after SNI. B7-H1 ko and WT mice did not differ in the number of entries **(C)** into the open arms after SNI. After SNI B7-H1 ko mice showed no alteration in the distance traveled **(D)**, whereas WT mice traveled less compared to naïve mice (*p* < 0.05 each). No intergroup difference was found after SNI. B7-H1 ko: young (8 weeks, naïve: 11 males, sham/SNI: 6 males/ group). WT: young (8 weeks, naïve: 11 males, sham/SNI: 6 males/ group). *p < 0.05.

In the LDB, no difference in anxiety-like behavior, as evaluated by time spent in the dark box, was detected between both genotypes in the naïve state and after SNI surgery (Figure 3A). Also, in the EPM, time spent in the closed arms and entries into the open arms did not differ between young B7-H1 ko and WT mice (Figures 3B,C). Exploratory behavior, as determined by the total distance traveled in the EPM, decreased in WT mice after SNI (*p* < 0.05, Figure 3D) without intergroup difference.

**Figure 4:**
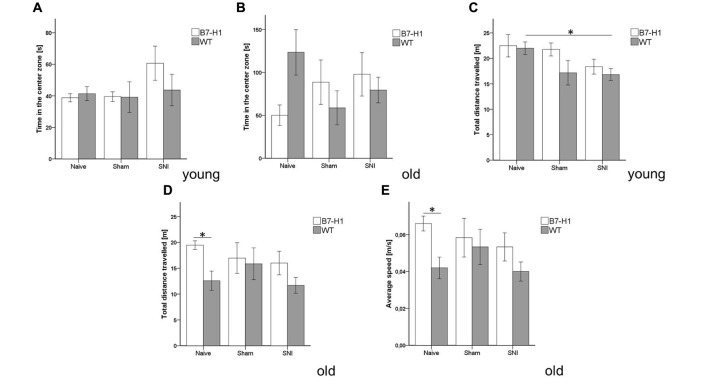
Anxiety-like behavior and locomotor activity in the open field (OF). Bar graphs show the results of the OF test. Male young (8 weeks) and old (12 months) B7-H1 ko and WT were investigated naïve, after sham surgery and after SNI. No difference between genotypes and surgeries was found in the time young **(A)** and old **(B)** mice spent in the center zone and in the total distance young B7-H1 ko mice traveled. Only WT mice traveled less after SNI compared to the naïve state (*p* < 0.05). **(C,D)** The total distance traveled was longer in old, naïve B7-H1 ko mice compared to WT mice. **(E)** Old, naive B7-H1 ko mice displayed a higher average speed compared to old, naïve WT mice. B7-H1 ko: young (8 weeks, naïve: 11 males, sham/SNI: 6 males) and old (12 months, 6 males). WT: young (8 weeks, naïve: 11 males, sham/SNI: 6 males) and old (12 months, 6 males /group). *p < 0.05.

Equally, in the complementary test for anxiety-like behavior using the OF, time spent in the center zone did not differ between genotypes, age-groups, and at baseline or after surgery (Figures 4A,B), except for a longer distance traveled by young naive WT mice compared to WT mice after SNI (*p* < 0.05, Figure 4C). Old naïve B7-H1 ko mice traveled a longer distance and displayed a higher average speed compared to naïve WT mice (*p* < 0.05 each, Figures 4D,E).

**Figure 5:**
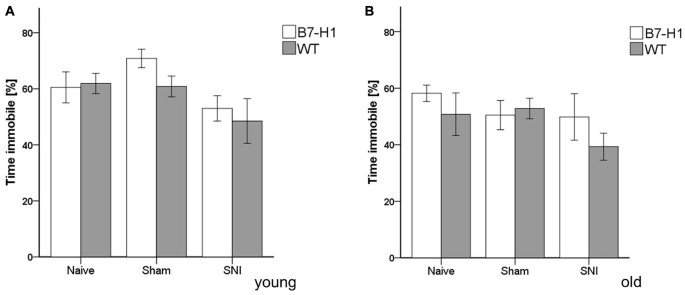
Depression-like behavior in the forced swim test (FST). Bar graphs show the results of depression-like behavior in the FST in young (8 weeks) and old (12 months), male B7-H1 ko and WT, naïve, after sham surgery and after SNI. The time spent immobile did not differ between genotypes and surgeries in young **(A)** and old **(B)** mice. B7-H1 ko: young (8 weeks, naïve: 11 males, sham/SNI: 6 males) and old (12 months, 6 males). WT: young (8 weeks, naïve: 11 males, sham/SNI: 6 males) and old (12 months, 6 males /group).

In the FST, young and old B7-H1 mice did not show any indications for depression-like behavior in the naïve state or after surgery (Figure 5).

### No Influence of SNI on Learning Behavior and Memory

**Figure 6:**
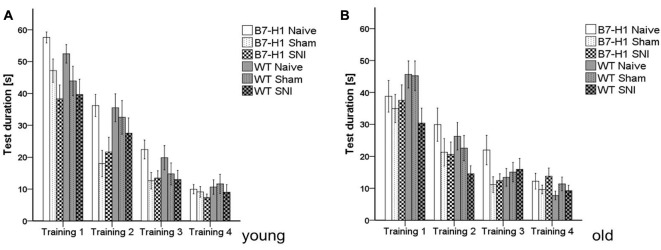
Cognitive behavior in the Morris water maze (MWM). Bar graphs show the results of the training in the MWM in young (8 weeks) and old (12 months), naïve, sham or SNI treated B7-H1 ko and WT were tested on four consecutive days. No difference between genotype and treatment was found in the time mice needed to reach the platform in young **(A)** and old **(B)** mice. B7-H1 ko: young (8 weeks, naïve: 11 males, sham/SNI: 6 males) and old (12 months, 6 males). WT: young (8 weeks, naïve: 11 males, sham/SNI: 6 males) and old (12 months, 6 males /group).

In both genotypes and age groups, the time mice needed to reach the hidden platform in the MWM was similar and did not change after surgery. Even the slope of the learning curves (i.e., decrease in test duration over time) did not differ between genotypes and age groups (Figure 6).

**Figure 7:**
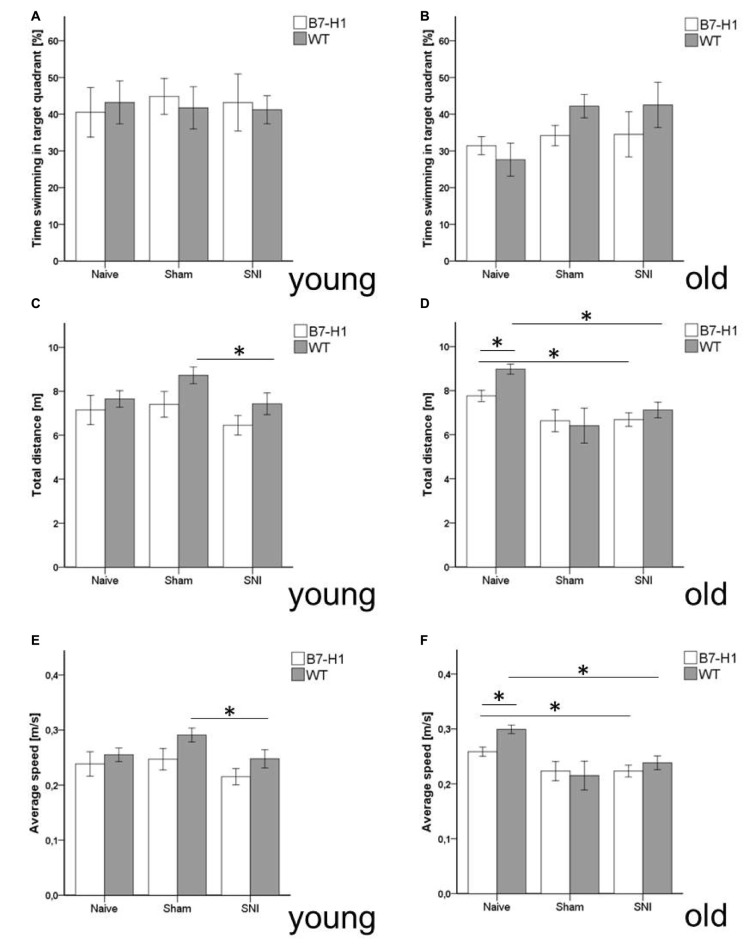
Memory and locomotor impairment in MWM. Bar graphs show the results of the probe trial (PT) in the MWM. Young (8 weeks) and old (12 months) B7-H1 ko and WT were investigated naïve, after sham and after SNI. No difference was found in the time swimming in the target quadrant between genotypes and surgery of young **(A)** and old mice. **(B,C)** Also, the total distance traveled did not differ in young B7-H1 ko and WT mice after SNI **(D)** The total distance traveled was shorter in old, naïve B7-H1 ko compared to WT mice (*p* < 0.05). Both genotypes traveled less after SNI (*p* < 0.05 each). **(E)** Average speed did not differ between genotypes and surgeries. **(F)** Average speed of naïve old B7-H1 ko mice was lower compared to naïve WT mice (*p* < 0.05). After SNI B7-H1 ko and WT mice were slower than naïve mice (*p* < 0.05). B7-H1 ko: young (8 weeks, naïve: 11 males, sham/SNI: 6 males) and old (12 months, 6 males). WT: young (8 weeks, naïve: 11 males, sham/SNI: 6 males) and old (12 months, 6 males/group). *p < 0.05.

Time spent swimming in the target quadrant did not differ between genotypes and age groups (Figures 7A,B), nor did the total distance swum by young mice of both genotypes before and after surgery (Figure 7C). Old naïve B7-H1 mice swam shorter distances than their WT littermates (*p* < 0.05, Figure 7D), while the further decrease in swimming distance after SNI did not differ from WT mice (*p* < 0.05 each, Figure 7D). While young mice of both genotypes did not differ in swimming velocity before and after SNI (Figure 7E), old naïve B7-H1 mice swam slower than their WT littermates (*p* < 0.05), however, a further decrease in swimming speed after SNI was similar in both genotypes (*p* < 0.05 each, Figure 7F).

### miR-21 Expression Is Increased 7 Days after SNI in the Injured Tibial and Common Peroneal Nerve of B7-H1 ko and WT Mice

No differences in *B7-H1* expression levels were detected in the sural nerve of WT mice between different age-groups (data not shown).

**Figure 8:**
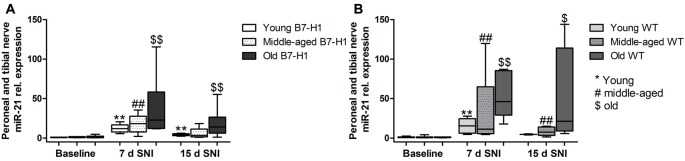
Relative gene expression of miR-21 in the tibial and common peroneal nerve. Boxplots show the miR-21 relative expression as measured by quantitative real-time-PCR (qRT-PCR) in young (8 weeks), middle-aged (6 months) and old (12 months) B7-H1 ko and wild-type littermate (WT) mice at baseline, 7 days and 15 days after SNI. **(A)** In young, middle-aged and old B7-H1 ko mice miR-21 was upregulated 7 days after SNI (*p* < 0.01 each). Young and old B7-H1 ko mice showed higher mir-21 expression levels 15 days (*p* < 0.01 each) after SNI. **(B)** In young, middle-aged and old WT mice miR-21 was upregulated 7 (*p* < 0.01 each) and 15 days (middle-aged: *p* < 0.01, old: *p* < 0.05) after SNI. Data were normalized to naïve WT mice. B7-H1 ko: young (naïve, *n* = 6; 7 days SNI, *n* = 6; 15 days SNI, *n* = 6), middle-aged (naïve, *n* = 6; 7 days SNI, *n* = 6; 15 days SNI, *n* = 6), old (naïve, *n* = 7; 7 days SNI, *n* = 6; 15 days SNI, *n* = 8). WT: young (naïve, *n* = 6; 7 days SNI, *n* = 6; 15 days SNI, *n* = 2), middle-aged (naïve, *n* = 6; 7 days SNI, *n* = 6; 15 days SNI, *n* = 6), old (naïve, *n* = 6; 7 days SNI, *n* = 6; 15 days SNI, *n* = 4). ^$^*p* < 0.05; **^,^^##,^^$$^*p* < 0.01.

miR-21 baseline expression levels did not differ in the tibial and common peroneal nerves of naïve B7-H1 ko and WT mice, and miR-21 levels were increased on day 7 after SNI in both genotypes without intergroup difference (*p* < 0.01 each, Figures 8A,B). miR-21 expression was also elevated on day 15 after SNI in both genotypes (young, old B7-H1 ko, and middle-aged WT: *p* < 0.01 each, old WT: *p* < 0.05, Figures 8A,B) except for middle-aged B7-H1 and young WT mice. No difference was detected in miR-21 expression between B7-H1 ko and WT mice of all age-groups on day 15 after SNI.

### After SNI miR-21 Is Upregulated Only in the Uninjured Sural Nerve of WT Mice

**Figure 9:**
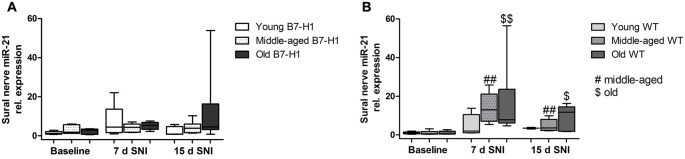
Relative gene expression of miR-21 in sural nerve. Boxplots show the miR-21 relative expression as measured by quantitative real-time-PCR (qRT-PCR) in young (8 weeks), middle-aged (6 months) and old (12 months) B7-H1 ko and wild-type littermate (WT) mice at baseline, 7, and 15 days after SNI. **(A)** Young, middle-aged and old B7-H1 ko mice did not show changes in miR-21 expression levels 7 and 15 days after SNI. In middle-aged mice B7-H1 mice showed a higher miR-21 baseline level in the sural nerve compared to WT mice (*p* < 0.05). **(B)** miR-21 was upregulated 7 (*p* < 0.01 each) and 15 (middle-age: *p* < 0.01, old *p* < 0.05) days after SNI in the sural nerve of middle-aged and old WT mice. Data were normalized to naive WT mice. B7-H1 ko: young (naïve, *n* = 6; 7 days SNI, *n* = 5; 15 days SNI, *n* = 6), middle-aged (naïve, *n* = 6; 7 days SNI, *n* = 5; 15 days SNI, *n* = 6), old (naïve, *n* = 7; 7 days SNI, *n* = 6; 15 days SNI, *n* = 8). WT: young (naïve, *n* = 6; 7 days SNI, *n* = 2; 15 days SNI, *n* = 2), middle-aged (naïve, *n* = 6; 7 days SNI, *n* = 6; 15 days SNI, *n* = 6), old (naïve, *n* = 6; 7 days SNI, *n* = 6; 15 days SNI, *n* = 5). ^$^*p* < 0.05; ^##,^^$$^*p* < 0.01.

**Figure 10:**
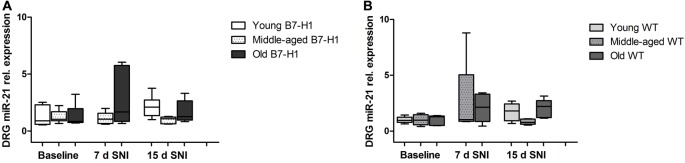
Relative gene expression of miR-21 in the dorsal root ganglia (DRG). Boxplots show the miR-21 relative expression as revealed by quantitative real-time-PCR (qRT-PCR) in young (8 weeks), middle-aged (6 months) and old (12 months) B7-H1 ko and WT littermate mice at baseline, 7 days, and 15 days after SNI. **(A)** miR-21 relative expression did not differ 7 days and 15 days after SNI in young, middle-aged and old B7-H1 ko mice. **(B)** Young, middle-aged and old WT mice did not show changes in miR-21 expression levels 7 and 15 days after SNI. Data were normalized to naive WT mice. B7-H1 ko: young (naïve, *n* = 6; 7 days SNI, *n* = 6; 15 days SNI, *n* = 6), middle-aged (naïve, *n* = 6; 7 days SNI, *n* = 6; 15 days SNI, *n* = 6), old (naïve, *n* = 7; 7 days SNI, *n* = 6; 15 days SNI, *n* = 8). WT: young (naïve, *n* = 6; 7 days SNI, *n* = 6; 15 days SNI, *n* = 6), middle-aged (naïve, *n* = 6; 7 days SNI, *n* = 6; 15 days SNI, *n* = 6), old (naïve, *n* = 5; 7 days SNI, *n* = 5; 15 days SNI, *n* = 5).

In the sural nerve of naïve, young B7-H1 ko, and WT mice miR-21 expression was not different between genotypes at baseline and did not change after surgery (Figure 9A). Middle-aged B7-H1 ko mice showed a 3-fold higher baseline miR-21 expression in the sural nerve compared to WT mice (*p* < 0.05) which did not change after surgery (Figures 9A,B), while miR-21 expression was increased on day 7, and 15 after SNI in middle-aged WT mice (*p* < 0.01 each, Figure 9B). In naïve old B7-H1 mice miR-21 expression was not different from WT littermates and did not change after surgery, however, in WT mice miR-21 expression was increased on day 7 after SNI (*p* < 0.01) and again showed a higher miR-21 expression compared to baseline values on day 15 (middle-aged: *p* < 0.01, old: *p* < 0.05, Figures 9A,B). There were no differences between genotypes of any age group, except for middle-aged WT mice displaying higher miR-21 expression levels than B7-H1 ko mice 7 days after SNI (*p* < 0.01). In summary miR-21 is upregulated after SNI in the sural nerve of WT mice, while B7-H1 ko mice seem to be spared. In the L4 and L5 DRG, miR-21 expression did not differ between naïve B7-H1 ko and WT mice at baseline and after surgery for all age-groups (Figure 10).

### SNI Induces Invasion of Macrophages and T Cells into the Injured Peroneal and Tibial Nerve

**Figure 11:**
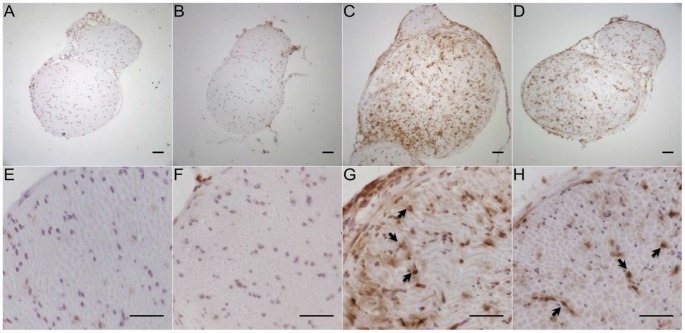
Representative photomicrographs of peroneal and tibial nerve frozen sections immunostained against CD11b for the detection of macrophages in young, naive B7-H1 ko **(A,E)**, and WT **(B,F)** mice, and 7 days after SNI (*n* = 4–5/genotype). Immunoreactivity for macrophages (arrows) was enhanced in B7-H1 ko **(C,G)** and WT **(D,H)** mice 7 days after SNI. Scale bars represent 50 μm. B7-H1 ko: young (8 weeks; naïve: 2 males, 3 females; sham: 3 males, 2 females; SNI: 5 males). WT: young (8 weeks; naïve: 3 males, 2 females; sham: 4 males, 1 females; SNI: 5 females).

**Figure 12:**
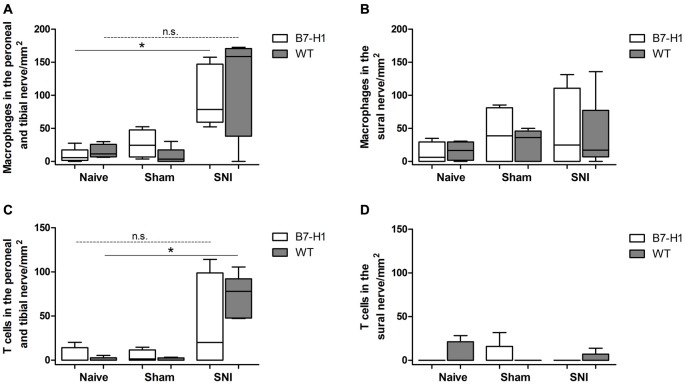
Quantification of the immunohistochemical staining of CD11b for the detection of macrophages and for the detection of T cells (CD3). **(A)** Increased number of macrophages in the tibial and common peroneal nerve of B7-H1 ko mice compared to naïve mice (*p* < 0.05). **(B)** No differences in number of macrophages in the sural nerve of B7-H1 ko and WT mice after SNI. **(C)** Increased number of T cells in the tibial and common peroneal nerve of WT mice after SNI (*p* < 0.05). **(D)** No differences in number of T cells in the sural nerve of B7-H1 ko and WT mice after SNI. B7-H1 ko: young (8 weeks; naïve: 2 males, 3 females; sham: 3 males, 2 females; SNI: 5 males). WT: young (8 weeks; naïve: 3 males, 2 females; sham: 4 males, 1 females; SNI: 5 females). *p < 0.05; n.s. not significant.

Immunohistochemistry with antibodies against CD11b for the detection of macrophages (Figure 11) in the tibial and common peroneal nerve of young B7-H1 ko and WT mice showed an increased number of macrophages 7 days after SNI in the tibial and common peroneal nerves of B7-H1 ko mice (*p* < 0.05), while WT mice only displayed a trend to higher numbers of macrophages (Figure 12A). The number of T cells also increased in the tibial and common peroneal nerve of WT mice (*p* < 0.05), whereas B7-H1 ko mice showed a trend to more T cells, which did not reach significance (Figure 12C). No differences were found in the number of macrophages and T cells in the sural nerve when comparing B7-H1 ko and WT mice in the naïve state and after SNI (Figures 12B,D).

## Discussion

We investigated a potential link between B7-H1 and miR-21 as key players in inflammation and neuropathic pain (Sheedy, 2015) and found a potential interplay between miR-21 induction in the tibial and common peroneal nerve after SNI and pain behavior.

Previously, we described an association between enhanced neural inflammation and sustained pain behavior in B7-H1 ko mice following chronic constriction injury (CCI; Üçeyler et al., 2010). To further characterize potential neuro-immune influences on the development and maintenance of pain and affective behavior, we applied the SNI model that leads to long lasting pain behavior (Decosterd and Woolf, 2000). We found mechanical hypersensitivity of young and middle-aged B7-H1 ko mice compared to WT mice at baseline. Differences in pain thresholds with ageing are in line with results of several studies reporting that age-dependent sensitivity in pain may develop inconsistently (Kim et al., 1995; Wang et al., 2006; Wang and Albers, 2009). Anatomical and functional changes, like reduction of nerve fibers and increased nerve damage with ageing (Chakour et al., 1996; Ceballos et al., 1999) may lead to higher nociceptive thresholds. Our results are in line with our previous study, where young B7-H1 ko mice also showed mechanical hypersensitivity (Üçeyler et al., 2010). In contrast to the CCI model, where B7-H1 ko mice showed sustained mechanical hyperalgesia after surgery, B7-H1 ko and WT mice of all age-groups developed mechanical hypersensitivity in the sural nerve territory without differences between genotypes after SNI. This may be due to the fact that pain behavior resolves within few weeks after CCI, while pain remains permanent after SNI (Decosterd and Woolf, 2000) and thus no recovery phase is reached in which differences between genotypes could be detected.

Chronic pain patients often report of anxiety, depression and cognitive impairment (Argoff, 2007; Moriarty et al., 2011). We therefore investigated anxiety and depression-like behavior in our mouse model before and after SNI. To cover the potential intra-individual variation and different facets of affective behavior, we performed three different exploration-based anxiety tests (Ramos, 2008). An influence of SNI on anxiety-like behavior was not detected in the performed tests, in any genotype or age-group. Previous studies reported increased anxiety in mice at later time points (30 days) after SNI in the LDB (Palazzo et al., 2016) and age-dependent increase in anxiety behavior (Lamberty and Gower, 1993; Boguszewski and Zagrodzka, 2002), which may be explained by the later time points investigated and different mouse strains used. To assess the influence of neuropathic pain on cognition, mice underwent the MWM test. Naïve, sham and SNI mice were equally good performers in the MWM training. These results are in line with studies in rats, displaying no influence of neuropathic pain on MWM test results (Leite-Almeida et al., 2009). Also, memory studied in the PT was not influenced by SNI surgery. In contrast to a study that identified young mice to be better performers than old ones (Francia et al., 2006), we also did not detect age differences.

Treating chronic neuropathic pain is a challenge and diagnostic biomarkers and new treatment options are warranted. miRNAs have been suggested as potential biomarkers in migraine, spinal cord injury and Parkinson’s disease (Andersen et al., 2016; Cosin-Tomás et al., 2016; Martirosyan et al., 2016). Since it is unclear, why individual patients are susceptible to developing neuropathic pain, whereas others are not, biomarkers for the identification of patients at risk would be helpful. miRNAs involved in cellular plasticity underlying neuropathic pain (Andersen et al., 2014) are thus promising candidates. Besides, silencing or mimicking an individual miRNA could be a potential strategy for treatment of neuropathic pain. We therefore explored the link between B7-H1 and miR-21, to gain new insight into this neuro-immune connection and its relevance for neuropathic pain. miR-21 is a downstream target of the B7-H1 receptor programmed-death 1 (PD-1). The lack of the PD-1 receptor causes enhanced binding of STAT5 in the miR-21 promotor area, which then leads to increased miR-21 expression. An upregulation of miR-21 has been shown in T cells of PD-1 ko mice (Iliopoulos et al., 2011). We used the B7-H1 ko mouse as a potential model of miR-21 overexpression, but did not detect miR-21 upregulation in the nervous tissue of our mice. One explanation might be that PD-1 has two ligands, B7-H1 and PD-L2 (Latchman et al., 2001). We speculate that a compensatory increase in PD-L2 expression might shift B7-H1 to subordinate relevance in miR-21 regulation.

**Figure 13:**
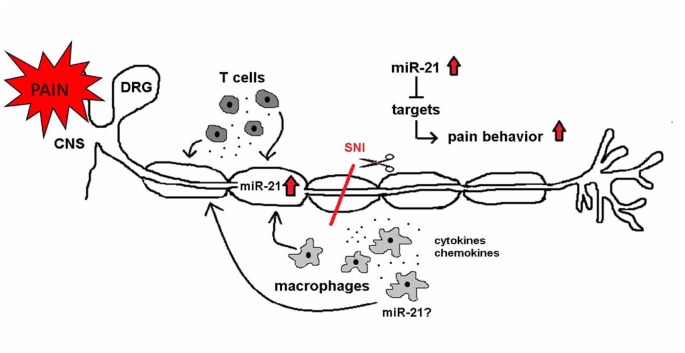
miR-21 as a potential mediator of neuropathic pain. After SNI miR-21 expression changes might influence neuronal functions and pain behavior. miR-21 is upregulated in the injured nerve. Immune cells (macrophages and T cells) infiltrate the injured nerve and are potential sources of miR-21. Additionally, immune cells release pro-inflammatory mediators (e.g., cytokines and chemokines), which contribute to neuropathic pain. Several targets of miR-21 influence neuronal associated pathways, and their inhibition is leading to pain.

miR-21 was the micro-RNA with the highest upregulation in mouse sciatic nerve after crush in a miRNA microarray (Wu et al., 2011). The same study showed an upregulation of several components of microRNA biosynthesis, like elements of the RISC complex and P-bodies (Wu et al., 2011), indicating a regulation of peripheral nerve regeneration processes by miRNA pathways. Others reported an upregulation of miR-21 in the spinal cord after CCI (Genda et al., 2013), suggesting a potential role of miR-21 in neuropathic pain. Furthermore, an upregulation of miR-21 was reported in the injured DRG after spinal nerve ligation and nerve resection in mice and rats (Strickland et al., 2011; Hori et al., 2016). Here, we show an upregulation of miR-21 in the injured tibial and common peroneal nerves of B7-H1 ko and WT mice after SNI surgery, and in the uninjured sural nerve only in middle-aged and old WT mice. These results suggest that miR-21 upregulation is related to the locus of injury and could also be associated with inflammation. We hypothesize that inflammatory cells may be the source of miR-21 (Figure 13), since macrophages infiltrate the peripheral nerve after axon injury and release cytokines and chemokines that contribute to peripheral sensitization (Calvo et al., 2012). Here, we found that immune cells infiltrated the injured tibial and common peroneal nerves of young B7-H1 ko and WT mice 7 days after surgery, while no changes were detected in the number of macrophages and T cells in the spared sural nerve. Another possibility for increased miR-21 levels could be that miR-21 is produced in the somata of the neurons and is transported along the axon as was shown for some miRNAs by others (Bicker et al., 2013; Ingoglia and Jalloh, 2016).

miR-21 upregulation might influence pain behavior by translational downregulation of multiple targets. The underlying mechanisms connecting miR-21 and pain are incompletely understood and only a few targets involved in nerve fiber sensitization and de- and regeneration have been described so far. One of these targets is the tumor suppressor phosphatase and tensin homolog (PTEN). A previous study identified PTEN as an important regulator of nociceptive behavior in a rodent model of neuropathic pain (Huang et al., 2015). Besides, miR-21 suppresses the production of tumor necrosis factor alpha (TNF) via PTEN (Sheedy, 2015) and inhibition of PTEN enhances outgrowth of adult peripheral axons (Yu et al., 2011), suggesting an additional role for PTEN in neuron sprouting. Another downstream target of miR-21 is programmed cell death 4 (PDCD4). A recent study showed that miR-21 induction in macrophages leads to a downregulation of PDCD4, which in turn results in an enhanced production of the anti-inflammatory cytokine interleukin-10 (IL-10; Das et al., 2014).

Our study has several limitations. We used the SNI model that induces severe and sustained pain behavior, thus, we may have missed mild intergroup differences. We restricted our analyses to miR-21, while many other miRNAs may be involved in neuropathic pain induction (Bali and Kuner, 2014; Norcini et al., 2014). Also, our data do not allow the cellular localization of miR-21 expression. Furthermore, contrary to expectations, expression levels of miR-21 did not differ between WT and B7-H1 ko mice limiting the relevance of the latter for miR-21 related pain. However, our data spot miR-21 as a promising candidate to determine potentially druggable downstream targets for future neuropathic pain treatment and add to the growing evidence on the crucial role of miRNAs in the regulation of neuro-immune circuits contributing to neuropathic pain.

## Author Contributions

FK: behavioral testing; data acquisition; data assessment and manuscript preparation. AG: data acquisition and manuscript preparation. NÜ and CS: study design; data assessment; manuscript preparation.

## Conflict of Interest Statement

The authors declare that the research was conducted in the absence of any commercial or financial relationships that could be construed as a potential conflict of interest.

## References

[B1] Andersen H. H. Duroux M. Gazerani P. (2014). MicroRNAs as modulators and biomarkers of inflammatory and neuropathic pain conditions. Neurobiol. Dis. 71, 159–168. 10.1016/j.nbd.2014.08.003 25119878

[B2] Andersen H. H. Duroux M. Gazerani P. (2016). Serum microRNA signatures in migraineurs during attacks and in pain-free periods. Mol. Neurobiol. 53, 1494–1500. 10.1007/s12035-015-9106-5 25636687

[B3] Argoff C. E. (2007). The coexistence of neuropathic pain, sleep and psychiatric disorders: a novel treatment approach. Clin. J. Pain 23, 15–22. 10.1097/01.ajp.0000210945.27052.b3 17277640

[B4] Bali K. K. Kuner R. (2014). Noncoding RNAs: key molecules in understanding and treating pain. Trends Mol. Med. 20, 437–448. 10.1016/j.molmed.2014.05.006 24986063 PMC4123187

[B5] Bicker S. Khudayberdiev S. Weiß K. Zocher K. Baumeister S. Schratt G. (2013). The DEAH-box helicase DHX36 mediates dendritic localization of the neuronal precursor-microRNA-134. Genes Dev. 27, 991–996. 10.1101/gad.211243.112 23651854 PMC3656329

[B6] Blyth F. M. March L. M. Nicholas M. K. Cousins M. J. (2003). Chronic pain, work performance and litigation. Pain 103, 41–47. 10.1016/s0304-3959(02)00380-9 12749957

[B7] Boguszewski P. Zagrodzka J. (2002). Emotional changes related to age in rats—a behavioral analysis. Behav. Brain Res. 133, 323–332. 10.1016/s0166-4328(02)00018-9 12110466

[B8] Brenner D. S. Golden J. P. Gereau R. W. IV (2012). A novel behavioral assay for measuring cold sensation in mice. PLoS One 7:e39765. 10.1371/journal.pone.0039765 22745825 PMC3382130

[B9] Calvo M. Dawes J. M. Bennett D. L. (2012). The role of the immune system in the generation of neuropathic pain. Lancet Neurol. 11, 629–642. 10.1016/S1474-4422(12)70134-5 22710756

[B10] Ceballos D. Cuadras J. Verdú E. Navarro X. (1999). Morphometric and ultrastructural changes with ageing in mouse peripheral nerve. J. Anat. 195, 563–576. 10.1046/j.1469-7580.1999.19540563.x 10634695 PMC1468027

[B11] Chakour M. C. Gibson S. J. Bradbeer M. Helme R. D. (1996). The effect of age on A delta- and C-fibre thermal pain perception. Pain 64, 143–152. 10.1016/0304-3959(95)00102-6 8867257

[B12] Chaplan S. R. Bach F. W. Pogrel J. W. Chung J. M. Yaksh T. L. (1994). Quantitative assessment of tactile allodynia in the rat paw. J. Neurosci. Methods 53, 55–63. 10.1016/0165-0270(94)90144-9 7990513

[B13] Chen G. Kim Y. H. Li H. Luo H. Liu D. L. Zhang Z. J.. (2017). PD-L1 inhibits acute and chronic pain by suppressing nociceptive neuron activity via PD-1. Nat. Neurosci. [Epub ahead of print]. 10.1038/nn.4571 28530662 PMC5831162

[B14] Cosin-Tomás M. Antonell A. Lladó A. Alcolea D. Fortea J. Ezquerra M.. (2016). Plasma miR-34a-5p and miR-545–3p as early biomarkers of Alzheimer’s disease: potential and limitations. Mol. Neurobiol. [Epub ahead of print]. 10.1007/s12035-016-0088-8 27631879

[B15] Crawley J. Goodwin F. K. (1980). Preliminary report of a simple animal behavior model for the anxiolytic effects of benzodiazepines. Pharmacol. Biochem. Behav. 13, 167–170. 10.1016/0091-3057(80)90067-2 6106204

[B16] Crisp T. Giles J. R. Cruce W. L. Mcburney D. L. Stuesse S. L. (2003). The effects of aging on thermal hyperalgesia and tactile-evoked allodynia using two models of peripheral mononeuropathy in the rat. Neurosci. Lett. 339, 103–106. 10.1016/s0304-3940(03)00009-0 12614905

[B17] Cunha F. Q. Poole S. Lorenzetti B. B. Veiga F. H. Ferreira S. H. (1999). Cytokine-mediated inflammatory hyperalgesia limited by interleukin-4. Br. J. Pharmacol. 126, 45–50. 10.1038/sj.bjp.0702266 10051119 PMC1565777

[B18] Das A. Ganesh K. Khanna S. Sen C. K. Roy S. (2014). Engulfment of apoptotic cells by macrophages: a role of microRNA-21 in the resolution of wound inflammation. J. Immunol. 192, 1120–1129. 10.4049/jimmunol.1300613 24391209 PMC4358325

[B19] Decosterd I. Woolf C. J. (2000). Spared nerve injury: an animal model of persistent peripheral neuropathic pain. Pain 87, 149–158. 10.1016/s0304-3959(00)00276-1 10924808

[B20] Dong H. Zhu G. Tamada K. Chen L. (1999). B7–H1, a third member of the B7 family, co-stimulates T-cell proliferation and interleukin-10 secretion. Nat. Med. 5, 1365–1369. 10.1038/70932 10581077

[B21] Dong H. Zhu G. Tamada K. Flies D. B. van Deursen J. M. Chen L. (2004). B7–H1 determines accumulation and deletion of intrahepatic CD8^+^ T lymphocytes. Immunity 20, 327–336. 10.1016/S1074-7613(04)00050-0 15030776

[B22] Ellis A. Bennett D. L. (2013). Neuroinflammation and the generation of neuropathic pain. Br. J. Anaesth. 111, 26–37. 10.1093/bja/aet128 23794642

[B23] Francia N. Cirulli F. Chiarotti F. Antonelli A. Aloe L. Alleva E. (2006). Spatial memory deficits in middle-aged mice correlate with lower exploratory activity and a subordinate status: role of hippocampal neurotrophins. Eur. J. Neurosci. 23, 711–728. 10.1111/j.1460-9568.2006.04585.x 16487153

[B24] Furtado M. Katzman M. A. (2015). Examining the role of neuroinflammation in major depression. Psychiatry Res. 229, 27–36. 10.1016/j.psychres.2015.06.009 26187338

[B25] Genda Y. Arai M. Ishikawa M. Tanaka S. Okabe T. Sakamoto A. (2013). MicroRNA changes in the dorsal horn of the spinal cord of rats with chronic constriction injury: a TaqMan^®^ low density array study. Int. J. Mol. Med. 31, 129–137. 10.3892/ijmm.2012.1163 23124577

[B26] Ha M. Kim V. N. (2014). Regulation of microRNA biogenesis. Nat. Rev. Mol. Cell Biol. 15, 509–524. 10.1038/nrm3838 25027649

[B27] Hargreaves K. Dubner R. Brown F. Flores C. Joris J. (1988). A new and sensitive method for measuring thermal nociception in cutaneous hyperalgesia. Pain 32, 77–88. 10.1016/0304-3959(88)90026-7 3340425

[B28] Hori N. Narita M. Yamashita A. Horiuchi H. Hamada Y. Kondo T.. (2016). Changes in the expression of IL-6-mediated microRNAs in the dorsal root ganglion under neuropathic pain in mice. Synapse 70, 317–324. 10.1002/syn.21902 26990296

[B29] Huang S. Y. Sung C. S. Chen W. F. Chen C. H. Feng C. W. Yang S. N.. (2015). Involvement of phosphatase and tensin homolog deleted from chromosome 10 in rodent model of neuropathic pain. J. Neuroinflammation 12:59. 10.1186/s12974-015-0280-1 25889774 PMC4386079

[B30] Iliopoulos D. Kavousanaki M. Ioannou M. Boumpas D. Verginis P. (2011). The negative costimulatory molecule PD-1 modulates the balance between immunity and tolerance via miR-21. Eur. J. Immunol. 41, 1754–1763. 10.1002/eji.201040646 21469086

[B31] Ingoglia N. A. Jalloh B. (2016). 76nt RNAs are transported axonally into regenerating axons and growth cones. what are they doing there? Neural Regen. Res. 11, 390–391. 10.4103/1673-5374.179035 27127463 PMC4828989

[B32] Jain R. Jain S. Raison C. L. Maletic V. (2011). Painful diabetic neuropathy is more than pain alone: examining the role of anxiety and depression as mediators and complicators. Curr. Diab. Rep. 11, 275–284. 10.1007/s11892-011-0202-2 21611765

[B33] Karam M. C. Al-Kouba J. E. Bazzi S. I. Smith C. B. Leung L. (2011). Interleukin-13 reduces hyperalgesia and the level of interleukin-1β in BALB/c mice infected with leishmania major with an up-regulation of interleukin-6. J. Neuroimmunol. 234, 49–54. 10.1016/j.jneuroim.2011.02.003 21402416

[B34] Kim Y. I. Na H. S. Yoon Y. W. Nahm S. H. Ko K. H. Hong S. K. (1995). Mechanical allodynia is more strongly manifested in older rats in an experimental model of peripheral neuropathy. Neurosci. Lett. 199, 158–160. 10.1016/0304-3940(95)12038-6 8584248

[B35] Kusuda R. Cadetti F. Ravanelli M. I. Sousa T. A. Zanon S. De Lucca F. L.. (2011). Differential expression of microRNAs in mouse pain models. Mol. Pain 7:17. 10.1186/1744-8069-7-17 21385380 PMC3060138

[B36] Lamberty Y. Gower A. J. (1993). Spatial processing and emotionality in aged NMRI mice: a multivariate analysis. Physiol. Behav. 54, 339–343. 10.1016/0031-9384(93)90120-5 8372130

[B37] Latchman Y. Wood C. R. Chernova T. Chaudhary D. Borde M. Chernova I.. (2001). PD-L2 is a second ligand for PD-1 and inhibits T cell activation. Nat. Immunol. 2, 261–268. 10.1038/85330 11224527

[B38] Leite-Almeida H. Almeida-Torres L. Mesquita A. R. Pertovaara A. Sousa N. Cerqueira J. J.. (2009). The impact of age on emotional and cognitive behaviours triggered by experimental neuropathy in rats. Pain 144, 57–65. 10.1016/j.pain.2009.02.024 19398158

[B39] Lemmer S. Schießer P. Geis C. Sommer C. Vanegas H. Üçeyler N. (2015). Enhanced spinal neuronal responses as a mechanism for the increased nociceptive sensitivity of interleukin-4 deficient mice. Exp. Neurol. 271, 198–204. 10.1016/j.expneurol.2015.06.011 26079835

[B40] Lewis B. P. Burge C. B. Bartel D. P. (2005). Conserved seed pairing, often flanked by adenosines, indicates that thousands of human genes are microRNA targets. Cell 120, 15–20. 10.1016/j.cell.2004.12.035 15652477

[B41] Lin C. R. Chen K. H. Yang C. H. Huang H. W. Sheen-Chen S. M. (2014). Intrathecal miR-183 delivery suppresses mechanical allodynia in mononeuropathic rats. Eur. J. Neurosci. 39, 1682–1689. 10.1111/ejn.12522 24612023

[B42] Martirosyan N. L. Carotenuto A. Patel A. A. Kalani M. Y. Yagmurlu K. Lemole G. M. Jr. (2016). The role of microRNA markers in the diagnosis, treatment and outcome prediction of spinal cord injury. Front. Surg. 3:56. 10.3389/fsurg.2016.00056 27878119 PMC5099153

[B43] Moriarty O. McGuire B. E. Finn D. P. (2011). The effect of pain on cognitive function: a review of clinical and preclinical research. Prog. Neurobiol. 93, 385–404. 10.1016/j.pneurobio.2011.01.002 21216272

[B44] Morris R. (1984). Developments of a water-maze procedure for studying spatial learning in the rat. J. Neurosci. Methods 11, 47–60. 10.1016/0165-0270(84)90007-4 6471907

[B45] Norcini M. Sideris A. Martin Hernandez L. A. Zhang J. Blanck T. J. Recio-Pinto E. (2014). An approach to identify microRNAs involved in neuropathic pain following a peripheral nerve injury. Front. Neurosci. 8:266. 10.3389/fnins.2014.00266 25221468 PMC4148822

[B46] Ostrand-Rosenberg S. Horn L. A. Haile S. T. (2014). The programmed death-1 immune-suppressive pathway: barrier to antitumor immunity. J. Immunol. 193, 3835–3841. 10.4049/jimmunol.1401572 25281753 PMC4185425

[B47] Palazzo E. Luongo L. Guida F. Marabese I. Romano R. Iannotta M.. (2016). D-aspartate drinking solution alleviates pain and cognitive impairment in neuropathic mice. Amino Acids 48, 1553–1567. 10.1007/s00726-016-2205-4 27115160

[B48] Pellow S. Chopin P. File S. E. Briley M. (1985). Validation of open: closed arm entries in an elevated plus-maze as a measure of anxiety in the rat. J. Neurosci. Methods 14, 149–167. 10.1016/0165-0270(85)90031-7 2864480

[B49] Porsolt R. D. Bertin A. Jalfre M. (1977). Behavioral despair in mice: a primary screening test for antidepressants. Arch. Int. Pharmacodyn. Ther. 229, 327–336. 596982

[B50] Prut L. Belzung C. (2003). The open field as a paradigm to measure the effects of drugs on anxiety-like behaviors: a review. Eur. J. Pharmacol. 463, 3–33. 10.1016/s0014-2999(03)01272-x 12600700

[B51] Ramos A. (2008). Animal models of anxiety: do i need multiple tests? Trends Pharmacol. Sci. 29, 493–498. 10.1016/j.tips.2008.07.005 18755516

[B52] Sakai A. Suzuki H. (2013). Nerve injury-induced upregulation of miR-21 in the primary sensory neurons contributes to neuropathic pain in rats. Biochem. Biophys. Res. Commun. 435, 176–181. 10.1016/j.bbrc.2013.04.089 23665014

[B53] Schoeniger-Skinner D. K. Ledeboer A. Frank M. G. Milligan E. D. Poole S. Martin D.. (2007). Interleukin-6 mediates low-threshold mechanical allodynia induced by intrathecal HIV-1 envelope glycoprotein gp120. Brain Behav. Immun. 21, 660–667. 10.1016/j.bbi.2006.10.010 17204394 PMC1991283

[B54] Sheedy F. J. (2015). Turning 21: induction of miR-21 as a key switch in the inflammatory response. Front. Immunol. 6:19. 10.3389/fimmu.2015.00019 25688245 PMC4310327

[B55] Shoji H. Takao K. Hattori S. Miyakawa T. (2016). Age-related changes in behavior in C57BL/6J mice from young adulthood to middle age. Mol. Brain 9:11. 10.1186/s13041-016-0191-9 26822304 PMC4730600

[B56] Soreq H. Wolf Y. (2011). NeurimmiRs: microRNAs in the neuroimmune interface. Trends Mol. Med. 17, 548–555. 10.1016/j.molmed.2011.06.009 21813326

[B57] Strickland I. T. Richards L. Holmes F. E. Wynick D. Uney J. B. Wong L. F. (2011). Axotomy-induced miR-21 promotes axon growth in adult dorsal root ganglion neurons. PLoS One 6:e23423. 10.1371/journal.pone.0023423 21853131 PMC3154476

[B58] Sun S. Chen D. Lin F. Chen M. Yu H. Hou L.. (2016). Role of interleukin-4, the chemokine CCL3 and its receptor CCR5 in neuropathic pain. Mol. Immunol. 77, 184–192. 10.1016/j.molimm.2016.08.006 27522478

[B59] Treede R. D. Jensen T. S. Campbell J. N. Cruccu G. Dostrovsky J. O. Griffin J. W.. (2008). Neuropathic pain: redefinition and a grading system for clinical and research purposes. Neurology 70, 1630–1635. 10.1212/01.wnl.0000282763.29778.59 18003941

[B60] Üçeyler N. Biko L. Hose D. Hofmann L. Sommer C. (2016). Comprehensive and differential long-term characterization of the alpha-galactosidase a deficient mouse model of fabry disease focusing on the sensory system and pain development. Mol. Pain 12:1744806916646379. 10.1177/1744806916646379 27145802 PMC4956180

[B61] Üçeyler N. Göbel K. Meuth S. G. Ortler S. Stoll G. Sommer C.. (2010). Deficiency of the negative immune regulator B7–H1 enhances inflammation and neuropathic pain after chronic constriction injury of mouse sciatic nerve. Exp. Neurol. 222, 153–160. 10.1016/j.expneurol.2009.12.026 20051242

[B62] Üçeyler N. Schäfers M. Sommer C. (2009). Mode of action of cytokines on nociceptive neurons. Exp. Brain Res. 196, 67–78. 10.1007/s00221-009-1755-z 19290516

[B63] Üçeyler N. Topuzoğlu T. Schiesser P. Hahnenkamp S. Sommer C. (2011). IL-4 deficiency is associated with mechanical hypersensitivity in mice. PLoS One 6:e28205. 10.1371/journal.pone.0028205 22164245 PMC3229527

[B64] Vale M. L. Marques J. B. Moreira C. A. Rocha F. A. Ferreira S. H. Poole S.. (2003). Antinociceptive effects of interleukin-4, -10 and -13 on the writhing response in mice and zymosan-induced knee joint incapacitation in rats. J. Pharmacol. Exp. Ther. 304, 102–108. 10.1124/jpet.102.038703 12490580

[B65] Wanet A. Tacheny A. Arnould T. Renard P. (2012). miR-212/132 expression and functions: within and beyond the neuronal compartment. Nucleic Acids Res. 40, 4742–4753. 10.1093/nar/gks151 22362752 PMC3367188

[B66] Wang S. Albers K. M. (2009). Behavioral and cellular level changes in the aging somatosensory system. Ann. N. Y. Acad. Sci. 1170, 745–749. 10.1111/j.1749-6632.2009.04011.x 19686222 PMC2844661

[B67] Wang S. Davis B. M. Zwick M. Waxman S. G. Albers K. M. (2006). Reduced thermal sensitivity and Nav1.8 and TRPV1 channel expression in sensory neurons of aged mice. Neurobiol. Aging 27, 895–903. 10.1016/j.neurobiolaging.2005.04.009 15979214 PMC2841704

[B68] Wu D. Raafat M. Pak E. Hammond S. Murashov A. K. (2011). MicroRNA machinery responds to peripheral nerve lesion in an injury-regulated pattern. Neuroscience 190, 386–397. 10.1016/j.neuroscience.2011.06.017 21689732 PMC3156291

[B69] Yu B. Zhou S. Qian T. Wang Y. Ding F. Gu X. (2011). Altered microRNA expression following sciatic nerve resection in dorsal root ganglia of rats. Acta Biochim. Biophys. Sin. 43, 909–915. 10.1093/abbs/gmr083 21908854

